# Strong preference of BRCA1 protein to topologically constrained non-B DNA structures

**DOI:** 10.1186/s12867-016-0068-6

**Published:** 2016-06-08

**Authors:** Václav Brázda, Lucia Hároníková, Jack C. C. Liao, Helena Fridrichová, Eva B. Jagelská

**Affiliations:** Institute of Biophysics, Academy of Sciences of the Czech Republic, v.v.i., Královopolská 135, 612 65 Brno, Czech Republic; School of Medicine, University of Queensland, Brisbane, 4006 Australia

**Keywords:** BRCA1 protein, DNA binding, Protein-DNA complex

## Abstract

**Background:**

The breast and ovarian cancer susceptibility gene *BRCA1* encodes a multifunctional tumor suppressor protein BRCA1, which is involved in regulating cellular processes such as cell cycle, transcription, DNA repair, DNA damage response and chromatin remodeling. BRCA1 protein, located primarily in cell nuclei, interacts with multiple proteins and various DNA targets. It has been demonstrated that BRCA1 protein binds to damaged DNA and plays a role in the transcriptional regulation of downstream target genes. As a key protein in the repair of DNA double-strand breaks, the BRCA1-DNA binding properties, however, have not been reported in detail.

**Results:**

In this study, we provided detailed analyses of BRCA1 protein (DNA-binding domain, amino acid residues 444–1057) binding to topologically constrained non-B DNA structures (e.g. cruciform, triplex and quadruplex). Using electrophoretic retardation assay, atomic force microscopy and DNA binding competition assay, we showed the greatest preference of the BRCA1 DNA-binding domain to cruciform structure, followed by DNA quadruplex, with the weakest affinity to double stranded B-DNA and single stranded DNA. While preference of the BRCA1 protein to cruciform structures has been reported previously, our observations demonstrated for the first time a preferential binding of the BRCA1 protein also to triplex and quadruplex DNAs, including its visualization by atomic force microscopy.

**Conclusions:**

Our discovery highlights a direct BRCA1 protein interaction with DNA. When compared to double stranded DNA, such a strong preference of the BRCA1 protein to cruciform and quadruplex structures suggests its importance in biology and may thus shed insight into the role of these interactions in cell regulation and maintenance.

**Electronic supplementary material:**

The online version of this article (doi:10.1186/s12867-016-0068-6) contains supplementary material, which is available to authorized users.

## Background

The BRCA1 protein is encoded by the tumor suppressor gene *BRCA1*, mutation in which occurs often in breast and ovarian cancer patients [1]. This multifunctional protein plays critical roles in different cellular pathways including cell cycle, transcription, DNA repair, DNA damage response and chromatin remodeling [2–4]. BRCA1 is a large phosphoprotein of 1863 amino acid residues (aa) and it is located primarily in cell nuclei. One of the key functions of BRCA1 protein is its ability to modulate multiple protein–protein and protein-DNA interactions. Despite the enormous molecular weight of BRCA1 protein, only two small conserved domains have been identified: ring finger motif (RING) at the N-terminus and two tandem BRCT repeats at the C-terminus. The central region of BRCA1 protein is largely unfolded, but it has been demonstrated to act as a scaffold to interacts directly with proteins and DNA [5]. It was determined that BRCA1 protein binds also to damaged DNA and regulates downstream target genes transcriptionally [6]. Moreover, previous studies have shown preferential binding of BRCA1 to cruciform [7], branch point [8] and superhelical [9] DNAs, highlighting the important relationship of BRCA1 protein with non-B DNA structures.

Non-B DNA structures are present in all living organisms [10] and are constantly been remodeled during processes such as DNA replication, transcription and repair. Local nucleotide sequence-dependent conformational changes, which give rise to cruciform, left-handed DNA, triplex and quadruplex structures, could all be stabilized further by negative supercoiling [11–13]. These non-B DNA structures can be recognized and stabilized also by various proteins, resulting in modulation of transcription [14], replication [15], junction resolving [16] and chromatin remodeling [17]. Cruciform structure, which originates from inverted repeats of variable length, plays key roles in replication and transcription [18, 19] and is a target for many essential proteins [20] including the human tumor suppressor proteins p53 [21, 22] and BRCA1 [23]. Triplex DNA, consisting of Watson–Crick and Hoogsten base-pairing, is formed by mirror repeats of homupurine-homopyrimidine sequences [24]. G-quadruplex DNA, as the name implies, arises from a G-rich sequence and forms a four-stranded structure through Hoogsteen base-pairing [25]. G-quadruplex structures were first characterized in vitro, but have nowadays been shown to exist in vivo using G-quadruplex stabilizing compounds [26] and specific G-quadruplex antibody [27]. The increased interest in G-quadruplexes stems from the high abundance of potential G-quadruplex-forming sequences in both eukaryotic and prokaryotic genomes (for reviews see ref: [28, 29]). In addition, the prevalence of G-quadruplexes in promoter regions and telomeres further reveals the significance of such structures in the genome.

It has been illustrated by microarray analysis that BRCA1 protein regulates the expression of a broad variety of genes [30]. Although upregulation of BRCA1 protein leads to drastic changes in transcription of gene targets, the mechanism remains unclear. Several studies have shown that the central region of BRCA1 is capable of interacting with DNA including short double-stranded oligonucleotide and long supercoiled DNA [7, 9, 23]. Additional findings further revealed BRCA1 protein’s selectivity for four-way junction DNA over linear duplex DNA [23, 31]. It is therefore likely that BRCA1 exerts its regulation by been able to recognize and bind DNA targets with different conformations directly.

Here we analyzed the binding of BRCA1 protein to various DNA targets with B- and non B-DNA conformations. Using gel shift assay, magnetic beads immunoprecipitation and atomic force microscopy (AFM), we demonstrated a strong preference of the central region (aa 444–1057) of the BRCA1 protein to non-B DNA structures, especially to cruciform and quadruplex DNA structures. Our findings further pointed to BRCA1 protein’s potential in regulating cellular processes by its direct interaction with DNA structures broadly present in the genomic DNA.

## Results

### BRCA1-L protein binds to different DNA targets

**Fig. 1:**
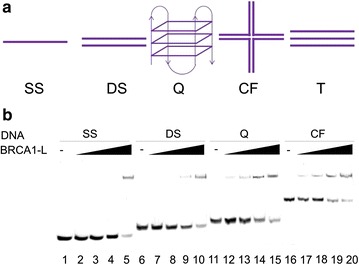
Comparison of BRCA1-L binding to different oligonucleotide structures. **a** Schematic representation of DNA substrates: *SS* single stranded, *DS* double stranded, *Q* quadruplex, *CF* cruciform, *T* triplex. **b** 5 pmol of labeled SS (*lane* 1–5), DS (*lane* 6–10), Q (*lane* 11–15), CF (*lane* 16–20), were incubated with increasing concentration of BRCA1-L (0/2.5/5/10/20 pmol) in the binding buffer (5 mM Tris–HCl, pH 7.0, 1 mM EDTA, 50 mM KCl and 0.01 % Triton X-100) for 15 min at 4 °C. Samples were electrophoresed on 8 % non-denaturating polyacrylamide gel at 100 V and 4 °C for 60 min

**Fig. 2:**
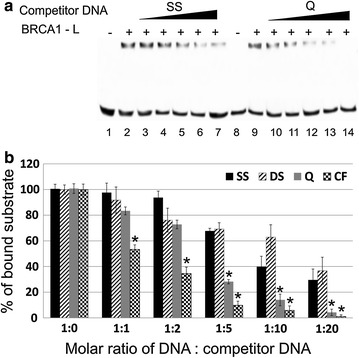
BRCA1-L competition assay. **a** Competition gel shift assay. 5 pmol of labeled CF was incubated with 5 pmol of BRCA1-L and increasing amount of competitor non-labeled DNA. Competitor DNAs on the image are *SS* single strand, *lane* 3–7 and *Q* quadruplex, *lane* 10–14. CF/Competitor DNA ratios were 1:1 (*lane* 3, 10), 1:2 (*lane* 4, 11), 1:5 (*lane* 5, 12), 1:10 (*lane* 6, 13), 1:20 (*lane* 7, 14). Samples were incubated 15 min on ice in the binding buffer and then loaded onto an 8 % non-denaturating polyacrylamide gel and electrophoresed for 90 min at 4 °C. *Arrow* shows localization of the BRCA1-L/DNA complexes. Complexes without competitor DNA (*lanes* 2, 9). **b** Graph representation of the competition assay. The relative intensity of the BRCA1-L/DNA complexes are expressed as the percentage of the bands without competitor DNAs. *Asterisks* denote statistically significant difference (p < 0.05) of BRCA1-L biding to non-B DNA versus DS

We analyzed the central region of BRCA1 protein (aa 444–1057: BRCA1-L, Additional file  1: Figure S1) binding to different DNA targets in detail. We prepared five different DNA oligonucleotide targets (Fig. 1a) that have implications in transcription and other cellular processes. Quality and purity of these structures were identified by their mobility in native polyacrylamide electrophoresis in 0.33× TBE buffer, and the formation of the G-quadruplex was confirmed by circular dichroism (CD) spectroscopy (data not shown). Due to structural changes of the DNA organization, the mobility of DS DNA was slower than that of SS DNA, as expected. The cruciform (CF) structure which is composed of 4 oligonucleotide strands displayed the slowest mobility followed by the quadruplex oligonucleotide of 51 bp. Formation of intermolecular triplex structure from oligonucleotide targets required the presence of magnesium ions (Mg^2+^) in the hybridization buffer as well in the polyacrylamide gel. Unfortunately, because the presence of MgCl_2_ inhibited the DNA-binding properties of BRCA1-L protein (not shown), gel-shift assay on PAGE gel was performed only for SS, DS, Q and CF DNA structures. As shown in Fig. 1b, BRCA1-L protein was able to bind to all the tested DNA substrates in the DNA binding buffer. Increased concentration of BRCA1-L protein (BRCA1-L:DNA molar ratio from 0.5:1 to 4:1) led to formation of retarded bands with slower mobility. At very low BRCA1-L/CF DNA molar ratios of 0.5:1 and 1:1, formation of retarded band was already evident (Fig. 1b, lanes 17, 18). Notably at molar ratio of 4:1, there was a near-complete disappearance of the free CF DNA (Fig. 2, lane 20) indicating that almost all the CF DNA has been bound by BRCA1-L protein. The gel shift assay also showed formation of retarded complexes with SS, DS and Q oligonucleotides, especially at high BRCA1-L/DNA molar ratio of 4:1 (Fig. 1b, lanes 5, 10, 15). However, lower BRCA1-L/DNA molar ratios led to formation of retarded band only with DS, Q and CF oligonucleotides. We did not observe any gel shift with BRCA1-A construct which lacks the DNA binding domain (aa 219–498, negative control, Additional file  1: Figure S2). Taken together, these results demonstrated that BRCA1-L, but not BRCA1-A protein, has a strong affinity to DNA even at low molar concentrations.

### Preferential binding of BRCA1-L protein to non-B DNA structures in short oligonucleotides on PAGE gel

To determine the preference of BRCA1-L protein to different non-B DNA structures, competition assay was performed. BRCA1-L protein was bound to FAM-labeled CF structure oligonucleotides with and without different competitor non-labeled DNAs (Fig. 2). Only a small decrease in retarded band intensity was observed with high concentrations of SS competitor DNA, while a stronger decrease was seen with lower concentrations of quadruplex competitor DNA (Fig. 2a). Using the same approach, we tested also competition of BRCA1-L/CF complex by DS and CF competitor DNAs. The change in intensity of retarded bands was analyzed by densitometry (Fig. 2b). SS and DS DNAs were weak binding targets for BRCA1-L protein compare to cruciform and quadruplex DNAs. Even 20-fold molar excess of SS or DS B-DNA competitor was not able to compete with BRCA1-L complex with cruciform structure (Fig. 2b, SS-black column, DS-dashed column). The strongest BRCA1-L-binding partner was cruciform structure (Fig. 2b, speckle column) followed by quadruplex oligonucleotide (Fig. 2b, grey column). While fivefold excess of SS or DS competitor DNA decreased retarded band intensity by approximately 30 %, cruciform and quadruplex competitor DNAs decreased retarded band intensity by around 90 and 72 %, respectively. Notably, a 20-fold surplus of CF and Q oligonucleotides led to completely ablation of retarded band intensity. Importantly, statistically significant difference (p < 0.05) between BRCA1-L binding to non-B DNA structures and DS was observed.

### Proof of the presence of non-B DNA structures in plasmid DNAs by atomic force microscopy

**Fig. 3:**
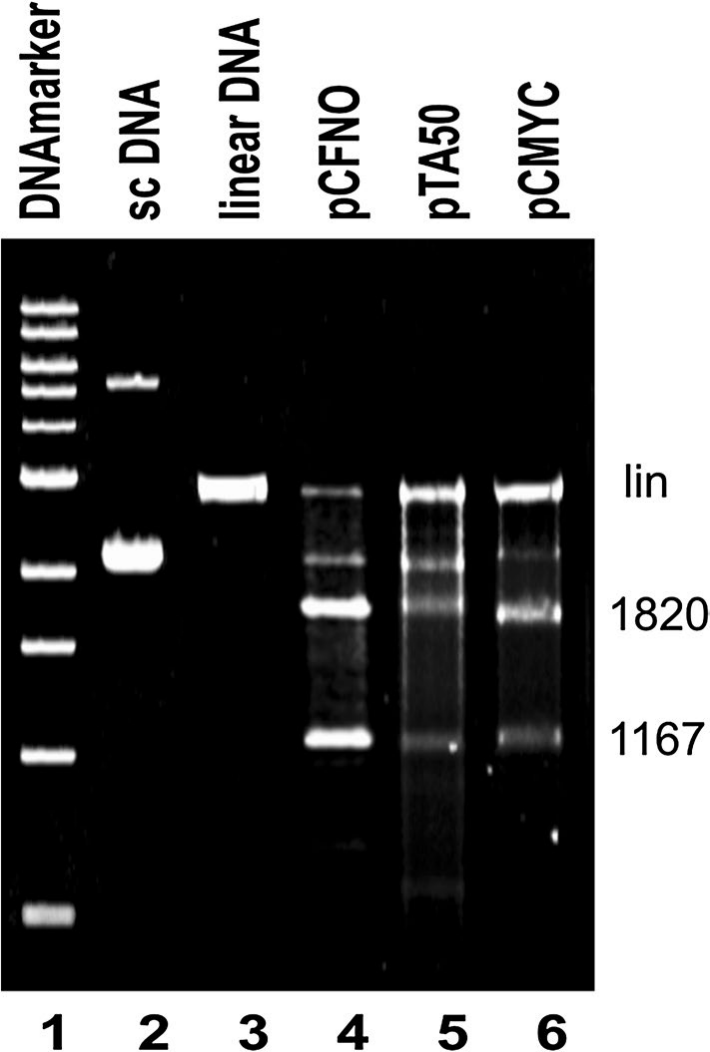
Evidence of non-B DNA structures in pCFNO, pCMYC and pTA50 plasmids by nuclease cleavage. The plasmids pCFNO (*lane* *4*), pCMYC (*lane* *6*) and pTA50 (*lane* *5*) have undergone cleavage by S1 nuclease with subsequent linearization by ScaI restriction endonuclease. Supercoiled pCFNO without any digestion (*lane 2*) and linearized pCFNO by *Sca*I (*lane* *3*) without S1 cleavage were used as controls. Bands resulting from the nuclease S1 cleavage are about 1820 and 1167 bp. *Lane* *1* contains the 500 bp DNA ladder

We used sequences that have the potential to form different non-B DNA structures in plasmid DNA. We documented in an earlier study that natural superhelical density in DNA could stabilize the formation of cruciform structure in plasmid pCFNO [21]. Moreover we employed plasmids pTA50 and pCMYC which are capable of forming intramolecular triplex and quadruplex, respectively (see “Methods” section). To confirm the presence and stabilization of these structures in superhelical DNA, we tested experimentally the presence of these structures within the plasmid DNA using nuclease S1 and *Sca*I cleavage. Our results showed a near-complete transition of inverted repeat into the cruciform structure in plasmid pCFNO, as suggested by practically total conversion of the plasmid DNA into the two fragments with size around 1167 and 1820 bp (Fig. 3a, lane 4). Similarly, we observed a strong cleavage in pCMYC plasmid, suggesting the presence of quadruplex structure in a large part of the plasmid with natural superhelical density (Fig. 3a, lane 6). The S1 cleavage with pTA50 (Fig. 3a, lane 5) revealed the presence of poorly defined fragments relative to the cleavage of pCFNO and pCMYC plasmids. However, clear bands at the anticipated lengths could still be localized. It is possible that this is associated with better accessibility of the longer SS DNA in the triplex structure by S1 nuclease. Nevertheless, it is clear that natural superhelical density is sufficient for triplex formation in at least part of the superhelical molecules.

**Fig. 4:**
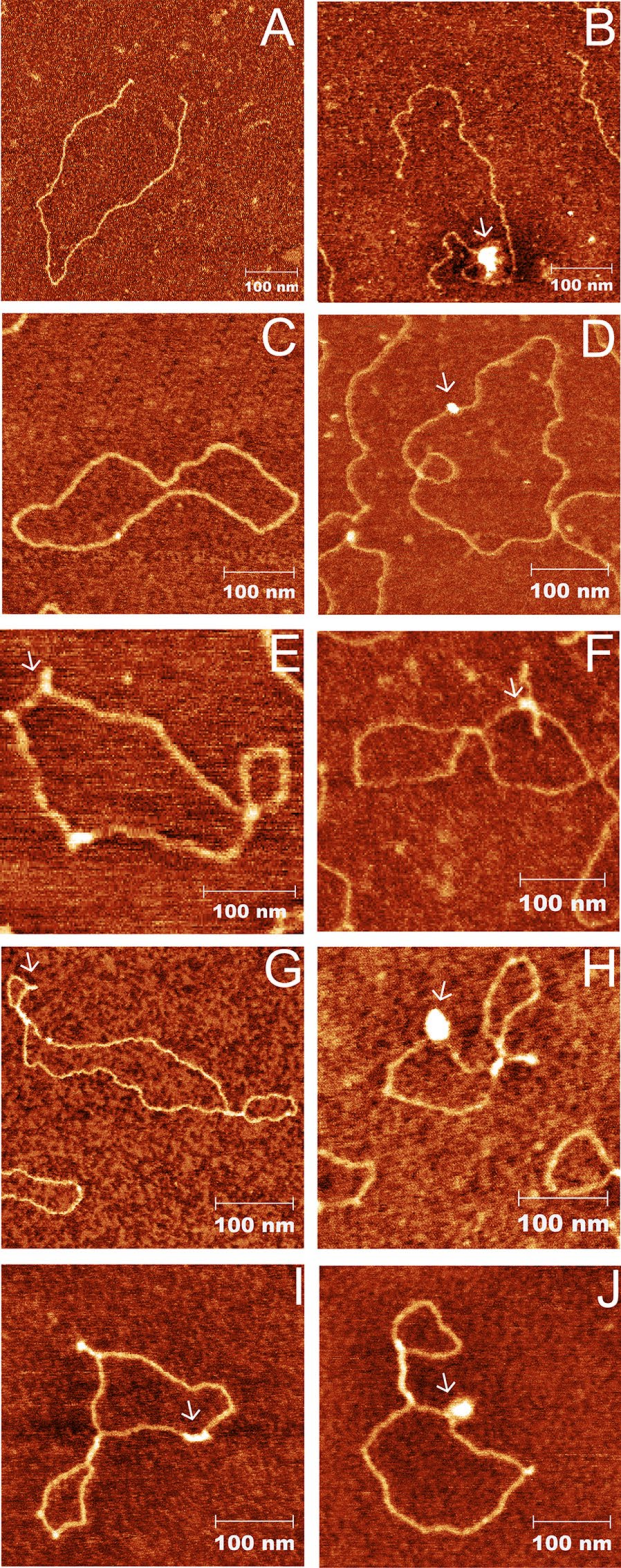
AFM images of DNA and BRCA1-L/DNA complexes. The first column shows the representative AFM images of different DNAs **A** linearized pBluescript, **C** superhelical pBluescript, **E** pCFNO containing cruciform structures (*arrow*), **G** pTA50 containing a triplex structure (*arrow*) and **I** pCMYC containing a quadruplex structure (*arrow*). In the second column **B**, **D**, **F**, **H**, **J**, the complexes of BRCA1-L with these plasmid DNAs are shown by arrows and are visible as *white circular spots* on the DNA strands

We investigated the binding of BRCA1-L protein to different plasmids with the potential to form non-B DNA structures. The presence of all structures in superhelical state was demonstrated also by AFM (Fig. 4). The AFM images displayed linear (Fig. 4A) and also superhelical (Fig. 4C) plasmid DNA of pBluescript. Using Gwyddion software for AFM image analyses, we measured the size of the observed local structures and analyzed at least 10 molecules per structure. Plasmid pCFNO containing inverted repeat in the *Hind*III site displayed local expanded structures visible as small extrusions of 0.96 ± 0.10 nm high and with variable length (Fig. 4E). Plasmid pTA50 often displayed structures of 16.9 ± 2.9 nm long and 0.98 ± 0.13 nm high corresponding to the length of triplex formation from d(A)_50_.d(T)_50_ sequence (Fig. 4G). Plasmid pCMYC showed formation of different structures including long blobs and spurs of 1.52 ± 0.10 nm high probably corresponding to quadruplex structures (Fig. 4I). The protein-DNA complexes were visualized under the same conditions as unbound DNA. BRCA1-L protein was able to bind to every kind of the DNA tested. The complexes of linear and supercoiled DNAs of pBluescript are shown in Fig. 4B and D, respectively. The frequency of BRCA1-L/DNA complexes differed among various types of DNA. However, in general, BRCA1-L protein formed complexes with linearized pBluescript least frequently than with supercoiled plasmids. Notably, supercoiled DNAs with non-B DNA structures were occupied by BRCA1-L protein more frequently than pBluescript. Interestingly, the location of BRCA1-L/DNA complexes is often seen in the cross-sections of DNA strands and at the extrusions of plasmids (Fig. 4H, J). Furthermore, the size of the complexes varies from the less commonly identified monomer to the more frequently observed large clusters, as demonstrated in Fig. 4B, H and J.

### Preferential binding of BRCA1-L protein to non-B DNA structures in long plasmid DNA

**Fig. 5:**
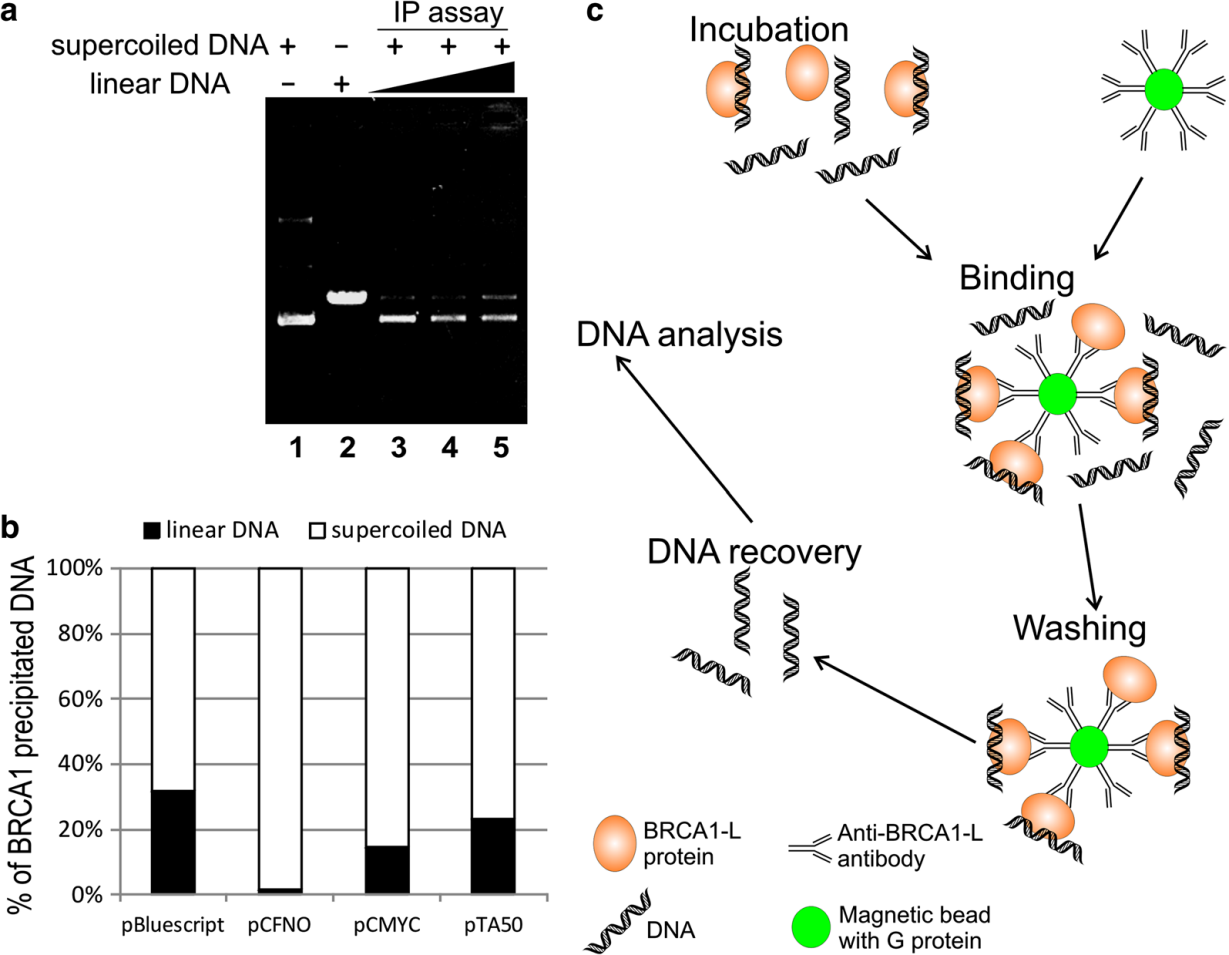
Comparison of the BRCA1-L protein binding to supercoiled and linear DNA by magnetic immunoprecipitation. **a** 0.3 μg superhelical pCFNO (*lanes* 3–5) and increased concentration of linear pCFNO/*Xho*I (0.3 μg—*lane* 3, 0.6 μg—*lane* 4, 1.2 μg—*lane* 5) were incubated with BRCA1-L protein (protein:DNA molar ratio 20:1) in binding buffer (5 mM Tris, 2 mM dithiothreitol 50 mM KCl, 0.01 % Triton X-100) with magnetic beads modified by the addition of BRCA1 monoclonal antibodies. BRCA1-DNA complexes were disrupted in a final step by incubation with 0.5 % SDS at 65 °C and then electrophoresed on 1 % agarose gel at 120 V and room temperature for 45 min. *Lanes* 1 and 2 contain control DNA of superhelical pCFNO and linear pCFNO/*Xho*I, respectively. **b** Graph representation of the BRCA1-L magnetic immunoprecipitation with different DNA targets. Ratio of the supercoiled and linear DNA before precipitation was 1:1. Densitometry of the DNA bands after immunoprecipitation with the BRCA1-L protein is shown in *bar*. **c** Scheme of the immunoprecipitation followed by DNA detection on magnetic beads

We analyzed BRCA1-L binding to DNA by gel shift analyses on gels with 0.33× TBE buffer. Because the mobility of linear and superhelical DNA in 1 % agarose gel with 0.33× TBE buffer is nearly identical, we used immunoprecipitation assay with magnetic beads, which is widely used in various assays including chromatin immunoprecipitation [35, 36], to compare BRCA1 protein binding to superhelical DNA (with non-B structure presented) and linear DNA (without non-B DNA structure presented). The BRCA1-L/DNA complexes were immobilized onto magnetic beads coated with protein G via the anti-BRCA1 antibody. After magnetic separation of the beads from the supernatant and washing, the BRCA1-L/DNA complexes were dissociated by heating in 0.5 % SDS and the recovered DNA was analyzed by agarose gel electrophoresis in 1 % agarose gel with 1× TAE buffer, in the absence of the BRCA1-L protein. Importantly, the mobility of superhelical and linear DNA differs under such conditions (Fig. 5, lanes 1 and 2). In agreement with gel shift analyses with oligonucleotides (Figs. 1, 2), our competitive immunoprecipitation assay consistently demonstrated a considerable strong preference of BRCA1-L protein for superhelical DNA with cruciform extrusion compared to linear DNA which is incapable of forming cruciform structure. As seen in Fig. 5 (lanes 3–5), even in the presence of relatively high abundance of linear DNA, BRCA1’s preference for superhelical DNA with cruciform extrusion is very strong as only very weak band of linear DNA was precipitated by the protein. According to the densitometry, almost all the DNA precipitated by BRCA1-L protein was supercoiled DNA with cruciform structure extrusion. In the molar presence of superhelical DNA, no more than 3 % of linear DNA was precipitated by BRCA1-L protein (Fig. 5a, lane 3). Notably, even abundance of the linear DNA (lin:sc ratio of 2:1, Fig. 5a, lane 4) did not change the BRCA1-L protein’s preference to superhelical DNA. Although the lin:sc ratio of 4:1 lead to increased precipitation of the linear DNA, sc DNA remained better precipitated by BRCA1-L protein (Fig. 5a, lane 5). The comparison of BRCA1-L affinities to different DNA structures in superhelical DNA is shown in Fig. 5b. In all cases, the supercoiled DNA was better precipitated by BRCA1-L protein than linear DNA. However, the ratio of the precipitated DNA differed according to the DNA structure presented in the plasmid DNA. The best target for BRCA1-L protein was pCFNO plasmid, followed by pCMYC, pTA50 and pBluescript. This result clearly revealed strong preference of the BRCA1-L protein to non-B DNA structures that are stabilized by superhelical stress.

## Discussion

BRCA1 is a multifunctional protein implicated in many important biological processes. It is a potent tumor suppressor and plays a major role in DNA repair and homologous recombination. BRCA1 protein is the most mutated gene in hereditary breast and ovarian cancers. Its mutation not only increased the lifetime risk of breast cancer to 65 %, but also increased the risk of other cancer types including prostate cancer [1]. It was shown that BRCA1 protein binds to DNA [7] and regulates transcription of specific proteins [30]. Strong preference of BRCA1 protein for cruciform structure has been demonstrated previously via gel shift assay on agarose gels [23]. It was also revealed that superhelical density could increase BRCA1 protein binding to DNA [9]. In this study we compared the binding of BRCA1 protein to DS DNA and non-B DNA structures and visualized these interactions using AFM. We showed a strong preference of BRCA1 protein for other non-B DNA structures such as quadruplex and triplex DNAs. Formation of non-B DNA structures is highly dependent on ion conditions, protein interactions and superhelical density of DNA. Magnesium ions are required for triplex DNA formation in oligonucleotide DNA, but they simultaneously inhibited BRCA1-DNA binding. Interestingly, we observed BRCA1 protein binding to DNA structures in plasmid DNA where these structures are stabilized by DNA supercoiling. Our results thereby demonstrated that native superhelical density is sufficient for non-B DNA structure formation. Furthermore, an array of experimental methods, including chromatin immunoprecipitation, confocal microscopy and functional assays, have illustrated that these structures are presented broadly in cells, with epigenetic modification being a potential mechanism of complex cell regulation. The presence of the magnesium could be an important factor which enables the formation of different DNA structures in cells [32, 33]. It was demonstrated that magnesium stabilizes DNA structures and plays a role in many enzymes’ catalytic action [34]. Even if the amount of magnesium in the cell is relatively high compare to other ions, the concentration of free magnesium is in fact low and most Mg^2+^ ions are bound to ATP, proteins and other cellular components [35]. Moreover, overexposure to magnesium is toxic [36]. It was also noted that bivalent ion influences the DNA binding of other protein greatly [37]. It is likely that tight regulation of the magnesium in the cell allows optimal BRCA1 DNA binding in living cells.

It has been revealed that BRCA1 protein plays a key role in homology-directed repair of DNA double strand breaks [38, 39] and facilitates end joining of DNA breaks [40]. Interestingly, certain local DNA structures could be the source of the DNA breaks [41]. Local DNA structures are known to facilitate different cellular processes including telomere length regulation, transcriptional modification, DNA replication and other events of cell maintenance. Hence, BRCA1’s ability to interact with these structures could be essential for cell survival and regulation. Over the last couple of years, it has brought to attention that non-B DNA structures, especially quadruplexes, are critical for transcriptional regulation of different genes including c-Myc proto-oncogene [14]. The presence of G-quadruplexes is also evident in many important gene promoters such as Kras, Kit and TERT [42] and a large number of proteins have been characterized with preferential binding to these quadruplexes [43]. It was reported that BRCA1 protein regulates telomerase and 3′ overhang length of telomeres [44]. Importantly, BRCA1 protein interacts directly with human telomeres. This is established by telomeric ChIP assay and confocal microscopy, showing co-localization of the BRCA1 protein with telomeric DNA in cultured cells [45]. Recently it was observed that BRCA1 mutation carriers have longer telomeres than their non-mutation carriers [46]. Moreover, BRCA1 is repeatedly absent or significantly decreased in sporadic breast cancer [47]. Given BRCA1 protein’s newly identified role in telomere regulation [45], its preferential binding to quadruplex DNA may indicate an important role in processes that are associated with quadruplex formation in the genome.

## Conclusion

It is well understood that BRCA1 protein binds to damaged DNA and plays a role in transcriptional regulation of downstream target genes. However, BRCA1-DNA binding properties to local DNA structures have not yet been reported in detail. Our study suggests a strong influence of non-B DNA structures on BRCA1-DNA interactions. These findings propose a novel perspective on the understanding of how BRCA1 protein regulates various tasks through direct interaction with DNA. The ability of BRCA1 protein to bind preferentially to topologically folded non-B DNA further hinted the value of these structures not only in transcriptional regulation, but also in processes leading to cancer development and senescence.

## Methods

### Synthetic oligonucleotides

Synthetic oligonucleotides with and without FAM-3′-end labeling were purchased from IDT, Inc. The oligonucleotide sequences and annealing buffers of single-stranded, double-stranded, cruciform, triplex and quadruplex DNAs are described in Additional file  1: Figure S3 (schema of DNA structures, Fig. 1a). Complementary oligonucleotides were annealed by incubation at 95 °C for 5 min with subsequent cooling to 4 °C at a rate of 1 °C/min. Oligonucleotide for quadruplex formation was incubated at room temperature for 16 h.

### Plasmid DNA

Supercoiled plasmid DNAs *of pBluescript II SK (−)*, and derived plasmids pCFNO [48], pCMYC and pTA50 were isolated from bacterial strain *DH5α* as described in the QIAGEN protocol (QIAGEN GmbH, Germany)*. Xho*I restriction enzyme (New England Biolabs, UK) was used for linearization of plasmids. pCMYC plasmid was constructed by cloning the 141 bp *Eco*RI/*Hind*III restriction fragment of pNHE plasmid [49] into the *Eco*RI/*Hind*III site of pBSK. pTA50 plasmid was constructed by cloning of (dT)_50_.(dA)_50_ sequence, forming a DNA triplex, into the *EcoR*V site of pBSK. Plasmids pCMYC and pTA50 were kindly provided by Dr. Marie Brazdova.

### BRCA1 protein constructs

The coding region for the central region of BRCA1 protein (BRCA1-A, aa 219–498 and BRCA1-L, aa 444–1057) was PCR amplified from human BRCA1 cDNA, subcloned into the pET15b expression vector (Novagen), expressed, and purified as described [9].

### Gel electrophoretic mobility shift assays on polyacrylamide gels

Labeled oligonucleotides (5 pmol) and BRCA1 protein constructs were mixed at different molar ratios (1:0.5/1/2/4) in 20 μl of the DNA binding buffer (5 mM Tris–HCl, pH 7.0, 1 mM EDTA, 50 mM KCl and 0.01 % Triton X-100). Competition assay contains increasing amount of competitor DNA (5/10/25/50 pmol) with 5 pmol of labeled cruciform oligonucleotide and 5 pmol of BRCA1-L protein in 20 μl of the DNA binding buffer. The samples were incubated for 15 min at 4 °C and loaded onto an 8 % non-denaturating polyacrylamide gel containing 0.33× Trisborate-EDTA buffer. Electrophoresis was performed for 60 min at 100 V at 4 °C. The gels were visualized on a LAS-3000 image analyzer (Fujifilm) and processed digitally.

### Statistical analysis

The relative intensity of the BRCA1-L/DNA complexes is presented as the percentage of the bands without competitor DNAs. Data were analyzed by non-parametric methods to avoid assumptions about the distribution of the measured variables. Comparisons between groups were made with the Mann–Whitney U test (Statistica software). All values are reported as mean ± SD. Statistical significance was considered to be indicated by a value of p < 0.05.

### Proof of the non-B DNA structures in plasmids by S1 nuclease cleavage

2 µg of plasmid DNA was digested by S1 nuclease (New England Biolabs, UK; 2 U/μg DNA) for 2 h at 37 °C in the S1 nuclease buffer (30 mM sodium acetate pH 4.6, 280 mM NaCl, 1 mM·ZnSO_4_). After digestion, samples were precipitated in ethanol, dissolved in water and digested by *Sca*I (New England Biolabs, UK) for 1 h at 37 °C before separation by electrophoresis on 1 % agarose gel.

### Atomic force microscopy

BRCA1-L protein and 200 ng of plasmid DNA were mixed in a molar ratio of 20:1 in the binding buffer [(50 mM KCl, 5 mM Tris, 0.05 mM EDTA, 0.01 % Triton X-100), final volume 10 μl] and incubated on ice for 15 min. AFM imaging was performed on Grade V4 mica discs (SPI supplies, USA). The DNA samples and protein-DNA complexes were deposited on mica in a buffer containing 5 mM Na-Hepes pH 7.5, 20 mM KCl, 10 mM MgCl_2_,10 mM Tris in the concentration of 1 ng/μl DNA and incubated for 5 min, followed by rinsing with deionized water and air-dried. The images were obtained using AFM/STM Multimode eight electrochemical system, (Veeco, USA), operating in ScanAsyst mode in room temperature in air. The cantilever SCANASYST-AIR (Bruker) had a nominal spring constant of 0.4 N/m and the nominal scanning rate was set as 1 Hz. Obtained images were then analyzed using Gwyddion software package [50].

### Competition assay by immunoprecipitation on magnetic beads

Superhelical and linear plasmids were incubated with protein G-coated magnetic beads (Dynabeads) using immobilized BRCA1-L immune complex with the anti-BRCA1 polyclonal antibody (Abcam) in the binding buffer. The samples were shaken gently for 30 min at 10 °C and then washed 3 times with the binding buffer. The BRCA-L/DNA complexes were disrupted by incubation with 0.5 % SDS for 5 min at 65 °C. The samples were loaded on a 1 % agarose gel containing 1x TAE (Tris–acetate-EDTA) buffer (Fig. 5).

## Additional file

10.1186/s12867-016-0068-6 SDS-PAGE of isolated BRCA1. **Figure S2.** Comparison of BRCA1-A binding to different oligonucleotide structures. **Figure S3.** Oligonucleotides used in the study.

## Abbreviations

aa: amino acid residues
AFM: atomic force microscopy
CD: circular dichroism
sc: supercoiled
BRCA1: breast cancer-associated protein-1
BRCA1-L: BRCA1-residues 444-1057
RING: really interesting new gene
BRCT: BRCA1 C-terminal
SS: single stranded
DS: double stranded
Q: quadruplex
CF: cruciform
T: triplex
