# Supplementary material for: Strong preference of BRCA1 protein to topologically constrained non-B DNA structures
Source: BMC Mol Biol. 2016 Jun 8;17:14. doi: 10.1186/s12867-016-0068-6 (PMC4898351; doi:10.1186/s12867-016-0068-6)
Supplement: Supplementary file 1 — 10.1186/s12867-016-0068-6 SDS-PAGE of isolated BRCA1. Figure S2. Comparison of BRCA1-A binding to different oligonucleotide structures. Figure S3. Oligonucleotides used in the study. [file 12867_2016_68_MOESM1_ESM.pdf]

Additional file 1

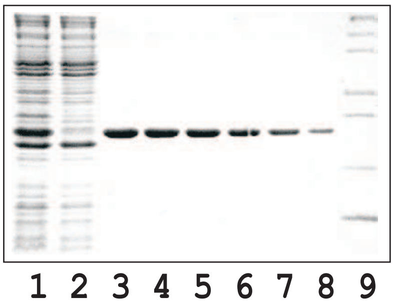

Figure S1: SDS-PAGE of isolated BRCA1.
Coomassie stained gel that depicts the quality and purify of BRCA1-L protein. Lysates of bacterial cells before (lane 2) and after (lane 1) IPTG induction, isolated BRCA1-L protein fractions (lanes 3-8), marker (lane 9).

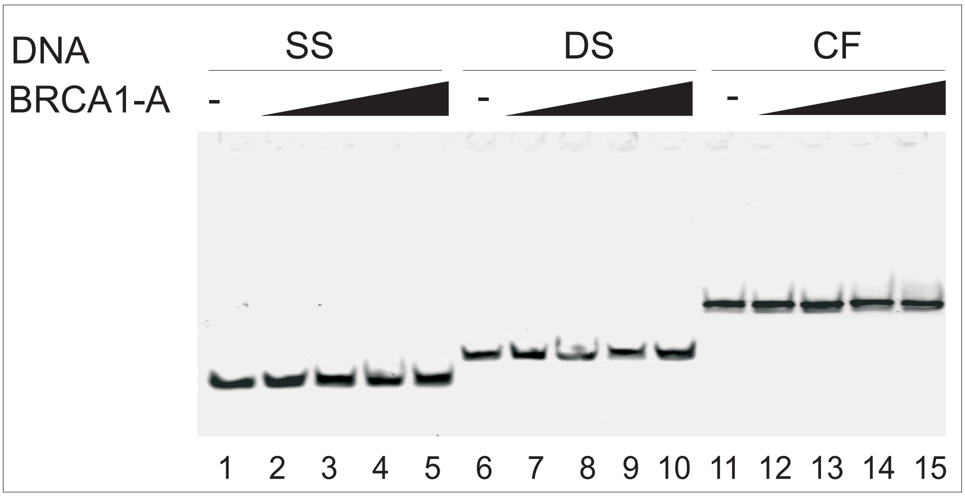

Figure S2: Comparison of BRCA1-A binding to different oligonucleotide structures.
5 pmol of labeled SS (lane 1-5), DS (lane 6-10), CF (lane 11-15) were incubated with increasing concentration of BRCA1-A (0/2.5/5/10/20 pmol) in the binding buffer (5 mM Tris-HCl, pH 7.0, 1 mM EDTA, 50 mM KCl and 0.01% Triton X-100) for 15 min at 4 °C. Samples were electrophoresed on 8% non-denaturating polyacrylamide gel at 100 V and 4 °C for 60 min.

Oligonucleotides used in the study

Abreviation	Structure	5´-3´sequence	Annealing buffer
**SS**	SS	CCCTCGGAGCACTGCACAACCCCTGGCCGCCG	AB1
**DS**	DS	CCCTCGGAGCACTGCACAACCCCTGGCCGCCG	AB2
		CGGCGGCCAGGGGTTGTGCAGTGCTCCGAGGG	
**CF**	CF	GAATTCAGCACGAGTCCTAACGCCAGATCT	
		AGATCTGGCGTTAGGTGATACCGATGCATC	
		CACTAGTCGTAAGCCACTCGTGCTGAATTC	
		CATGCATCGGTATCAGGCTTACGACTAGTG	AB3
**Q**		GGGTTAGGGTTAGGGTTAGGGTTAGGGTTAGGGTTAGGGTTAGGGTTAGGG	AB3

AB1: 10 mM Tris, 1mM EDTA, pH 7,8

AB2: 100 mM sodium phosphate , 150 mM NaCl, 1mM EDTA, pH 8

AB3: 50 mM KCl, 5 mM Tris, 0,05 mM EDTA, 0,01% Triton X - 100

Figure S3: Oligonucleotides used in the study
